# Factors encouraging participation in social activities after hospital discharge in people with severe mental illness who received occupational therapy

**DOI:** 10.3389/fpsyt.2024.1421390

**Published:** 2024-08-26

**Authors:** Izumi Nagashima, Tomonari Hayasaka, Koji Teruya, Miku Hoshino, Masami Murao, Yasuyuki Matumoto, Taku Maruki, Takeshi Katagiri, Yayoi Imamura, Mariko Kurihara, Yuki Oe, Takashi Tsuboi, Koichiro Watanabe, Hitoshi Sakurai

**Affiliations:** ^1^ Department of Rehabilitation, Kyorin University Faculty of Health Sciences, Tokyo, Japan; ^2^ Kyorin University Faculty of Health Sciences, Tokyo, Japan; ^3^ Kyorin University Hospital, Tokyo, Japan; ^4^ Department of Neuropsychiatry, Kyorin University Faculty of Medicine, Tokyo, Japan

**Keywords:** occupational therapy, psychiatric rehabilitation, recovery, severe mental illness, social activities

## Abstract

**Introduction:**

Occupational therapy (OT), a vital part of psychiatric rehabilitation, encourages participation in social activities, which is critical for the recovery of people with severe mental illnesses (SMI). However, the effects of OT on the subsequent social activities of patients with SMI have not been fully clarified. We aimed to identify the factors that encourage post-discharge social activity participation among patients with SMI who received OT.

**Method:**

Patients who underwent OT at the Kyorin University Hospital between April 2016 and March 2020 were retrospectively examined for baseline data during hospitalization and social activity status 1 year after discharge. Occupational support, group adaptation, artistic activities, and exercise programs were considered. Activities requiring social interaction were defined as social activities, including employment, schooling, sheltered work, and volunteer work. Multiple logistic regression analyses using demographic and medical data, prehospitalization social activity status, and OT participation rates as independent variables were used to examine the factors encouraging social activity participation after discharge. Decision tree analysis was conducted to identify patients who specifically needed to increase OT participation.

**Results:**

Of 524 eligible patients, 247 were included in the study. The number of patients who were socially active at admission and after discharge was 116 and 188, respectively. Multiple logistic regression analyses revealed that the following factors were likely to encourage social activity participation after discharge: higher rates of OT participation to facilitate group adaptation (OR = 1.015, 95% CI 1.003–1.027), being socially active at admission (OR = 4.557, 95% CI 2.155–9.637), and no marital history (OR = 0.293, 95% CI 0.130–0.661). Decision tree analysis showed that for patients who were socially inactive at admission and had a history of marriage, increasing OT participation to 52.6% or higher may ensure social activity participation after discharge.

**Conclusions:**

This study identified patients whose social participation after discharge could be boosted by OT that facilitates group adaptation. Our findings would facilitate the development of individualized add-on rehabilitation based on the effects of real-world OT practices.

## 1 Introduction

People with severe mental illnesses (SMI) often find themselves distanced from the social activities they wish to join or those suggested by others (1–4). They typically require support to reintegrate into social activities, especially after hospitalization. Building connections through these activities is essential for personal recovery (5). Moreover, procuring employment — a crucial form of community involvement — enhances the quality of life of people with SMI (6, 7) and can help reduce healthcare costs by potentially preventing rehospitalization (8). However, people with SMI, who are prone to social withdrawal or disconnection, often face challenges re-engaging in social activities after hospital discharge (9–11). Consequently, consistent mental health treatment is paramount to bridging their return to social activities, although many tend to discontinue such treatment (12). Such adverse outcomes are more likely to occur in the period immediately following hospital discharge. In a systematic review, Mutschler et al. (13) identified that discharged patients experienced seven shortfalls, including “self-care as a coping strategy” and unfavorable “external factors.” “Self-care as a coping strategy” included “meaningful activity” and voluntary work, hobbies, making social contacts, and returning to work. However, this study showed that patients experience difficulties searching and finding these activities.

In-patient rehabilitation plays a crucial role in supporting people with SMI to continue mental health treatment and rejoin social activities. Killaspy et al. (14) conducted a 12-month prospective observational study with a cohort of patients admitted to quality-assured rehabilitation units in England. The study included 329 patients, 68% of whom were diagnosed with schizophrenia. During the study period, 70% of patients were either discharged or ready for discharge, with evidence of an associated reduction in cost of care. Similarly, Bunyan et al. (15) conducted a retrospective study with a cohort of patients from three in-patient rehabilitation units. The study involved 22 patients, including 13 patients with schizophrenia. The researchers found that the number of admission days and bed costs significantly decreased over a 2-year period following admission to the in-patient rehabilitation units, compared to that prior to admission. Guidelines from the National Institute for Health and Care Excellence emphasize the importance of staff attitudes and the structure of programs concerning recovery-oriented rehabilitation services for complex mental states (16).

Occupational therapy (OT) is an integral part of mental health rehabilitation services. Two systematic reviews have shown that there is strong evidence for the community reintegration of patients with SMI and the improvement of symptoms in patients with depression in an OT program structure aimed at return to work (17, 18). Additionally, the efficacy of OT in aiding people with SMI, especially schizophrenia, has been well documented, from improving psychiatric symptoms and cognitive functions to enhancing patient quality of life and interpersonal skills (19–22). These studies have highlighted the impact of OT on specific functions in patients with chronic schizophrenia who were hospitalized for an average of over 5 years. Research on patients with an average hospital stay of less than three months emphasized the importance of meaningful occupation in ensuring a positive hospital experience and successful reintegration post-discharge (23). In addition, people with schizophrenia who participated in individual OT during hospitalization continued to improve their social functioning for 5 years after discharge (24). A scoping review of OT in acute care psychiatric short-term hospitalization units showed that group interventions and spiritual aspects had a positive impact on quality of care and meaningful daily life for patients (25). However, these studies did not provide specific outcomes after discharge. Moreover, while there are many reports showing the effectiveness of OT as an add-on intervention implemented in a research setting, there is limited understanding of the effectiveness of real-world OT practices implemented at baseline.

A specific outcome after discharge is a compelling fact of the effect of the intervention performed during hospitalization. There is a gap between OT practices during hospitalization that support a positive inpatient experience, alleviate psychiatric symptoms, improve cognitive and social functioning, and provide a spiritual aspect, and those that keep the patient active in society after discharge. For real-world OT practice with inpatients to truly contribute to patients’ social activity, it is necessary to investigate real-world OT factors related to specific outcomes after discharge. To further develop the individualized add-on OT programs described in previous studies, it is necessary to identify the characteristics of patients with SMI who need enhanced real-world OT.

Therefore, we aimed to evaluate the post-discharge outcomes of patients with SMI who underwent OT during hospitalization in an acute care university hospital. Notably, we examined their participation in social activities within 1 year of discharge to determine the factors that promoted social activity and the hierarchy among the factors, and to identify patients with SMI for whom OT practices should be intensified.

## 2 Materials and methods

### 2.1 Patients

This was a retrospective analysis of post-discharge outcomes for patients with SMI who engaged in OT programs during hospitalization at an acute care university hospital. In this study, patients with SMI were defined as patients with psychiatric disorders requiring acute inpatient care (8). Patients admitted to the Department of Neuropsychiatry at Kyorin University Hospital between April 2016 and March 2020 who received OT were included in this study. The exclusion criteria were: (a) the patient was not admitted for treatment purposes but for examination to confirm their psychiatric diagnosis and (b) the patient was transferred to other facilities without any follow-up visits to our hospital; in other words, all patients included in this study attended our outpatient department after discharge. A portion of this sample was included in a previous study on employment attainment factors in people with mood disorders who participated in an occupational support program (26). This study was approved by the Ethics Review Committee of Kyorin University School of Medicine (Approval number: R03-095).

### 2.2 Occupational therapy for mental disorders

The OT programs were conducted in group sessions with approximately 10 participants and two occupational therapists. The programs were conducted according to patients’ conditions, treatment goals, and personal preferences. Consequently, the initiation timing of the program varied between participants. Therefore, the demographic compositions of the OT groups were diverse and uncontrolled regarding the diagnosis of psychiatric disorders, gender, or age. The OT programs comprised four types, each administered once a week in a 2-hour session: occupational support program (OSP) (26), seasonal activity program (SAP), artistic activity program (AAP), and exercise program (EP).

The primary aim of the OSP was to evaluate participants’ cognitive function and work performance, aiming to enhance the competencies they would need for rejoining the workforce or resuming academic pursuits (26). As cognitive tasks, patients played a bingo game in which they were asked to name the words they could think of that corresponded to a given theme, and solved a puzzle of geometric figures. Patients were then asked to transcribe a newspaper in timed segments and count the number of transcribed letters and misspelled words to encourage self-monitoring of their fatigue level. This program was designed as an independent activity so that it could be established even if patients participated only once. Patients who continued to participate were asked to draw a graph of the number of letters they transcribed and their level of fatigue to visualize the changes.

The main objective of the SAP was to foster group adaptation. The key activities included walking through the hospital garden and engaging in seasonal crafts. During the walk, everyone walked at the pace of the patients with the least walking ability, taking their time to enjoy the seasonal flowers, plants, and trees. Seasonal crafts were performed by all patients; one paper picture was to be completed approximately six times, comprising one session. Depending on the patient’s state of recovery, their roles consisted of a single task, such as cutting paper with their fingers, cutting paper with a tool, or pasting paper onto the mount. Patients who were easily fatigued because their severe psychiatric symptoms had just subsided were allowed to simply be present without taking on an active role. In addition, to facilitate interaction among the patients, staff members reflected on the walk and brought up the topic of the design of the mount.

The AAP aimed to assess and bolster participants’ executive function and occupational performance while encouraging the cultivation of interests. Its core component allowed participants to prepare and engage in activities selected from a pool of over 20 options tailored to their individual schedules, thus ensuring personalized participation. Optional activities were those that could be completed in a minimum of one session and a maximum of four sessions, including traditional origami, coloring, scratch painting, sewing, leatherworking, cord-working, and tile mosaic, among others. The tools and materials needed for the activities were placed in one corner of the room, and patients had to prepare and clean up on their own and offer help to the staff if they did not understand. In addition, patients introduced themselves to the group before the activity began and explained the activity they had performed at the end.

The EP-integrated group underwent stretching and paired light circuit training. The objectives of this module were twofold: to sustain and enhance participants’ physical well-being and stimulate interpersonal engagement. The program was set up independently so that it could be established even if a patient only attended one session, and the content was the same each time. Stretching was performed in a chair-sitting position, with one of the occupational therapists positioned relative to the patients as the leader, and the patients imitated the leader’s movements. The following items were arranged for circuit training: a 15-cm step stool going up and down, a 3-kg ball delivered backward by trunk rotation, flexion and extension of the upper limbs with bands of varying strength, and air cushion standing and foot stomping. Patients paired up, counted each other’s steps in the step up and down, passed the ball to their partner according to their trunk rotation limits, adjusted the band to match their partner’s ability, and helped their partner beside the air cushion to prevent them from falling over.

### 2.3 Study design and survey contents

In this study, we retrospectively examined medical and OT records. For patients who were hospitalized more than once during the study period, information was obtained from their last admission. We collected the following demographic and clinical variables at baseline: gender, age at discharge, academic degree, marital history, social activity status at admission, diagnosis of psychiatric disorder, duration of psychiatric disorder, Global Assessment of Functioning (GAF) scores, number of psychotropic types, admission period, and number of past hospitalizations. The participation ratio of each OT program relative to patients’ length of hospitalization was recorded. Diagnoses of psychiatric disorders were categorized as follows: neurodevelopmental disorders (e.g., intellectual disability, autism spectrum disorder, attention-deficit/hyperactivity disorder), schizophrenia spectrum (e.g., delusional disorder, brief psychotic disorder, schizophrenia, schizoaffective disorder), mood disorders (e.g., bipolar and related disorders, depressive disorders), major neurocognitive disorders (e.g., various dementias), and others (e.g., anxiety disorders, obsessive-compulsive and related disorders, trauma- and stressor-related disorders, dissociative disorders, somatic symptom and related disorders, feeding and eating disorders, sleep-wake disorders, substance-related disorders, personality disorders). The primary outcome was engagement in social activities within 1 year post-discharge. Each patient’s social activity status (at admission or within 1 year post-discharge) was identified from the psychiatrist’s description of the patient during the interview (at the inpatient department or outpatient department). Because there were patients who commuted to work or school from the psychiatric ward—to ensure that they would remain in good standing at work or school prior to discharge—and the dates on which patients described their participation in social activities were not strictly the dates on which they had participated in them, the number of days to achieve participation in social activities was not investigated. Social activities were defined as employment or academic pursuits, job seeking or utilization of social services (27), self-employment or farming, and voluntary roles or hobbies necessitating interactions and negotiations with others. Based on these criteria, the patients were divided into two groups: those who implemented social activities during the first year after discharge (social activities group) and those who did not (non-social activities group).

### 2.4 Statistical analysis

Demographic, clinical, and OT-related variables were presented using descriptive statistics. Multiple logistic regression analysis was used to identify factors predicting post-discharge social activity engagement. We preliminarily analyzed descriptive statistical variables and incorporated gender, age, academic degree, marital history, GAF score, duration of disorders, social activity status at admission, and participation rates in the four OT programs into a stepwise selection process for the multiple logistic regression analysis. For the variable entry method, forced entry was used for the diagnosis of psychiatric disorders, while a stepwise selection method was applied for other variables exhibiting a potential difference (p < 0.5) between the social activity and non-social activity groups. Subsequently, a decision tree analysis utilizing the classification and regression tree (CART) algorithm was conducted to elucidate the hierarchical interrelationships of factors influencing post-discharge social activity status and identify patient groups that needed increased OT participation. CART aims to discern mutually exclusive subgroups within a population sharing characteristics that influence a particular dependent variable and demands the preliminary determination of independent variables (28). In the decision tree analysis, the dependent variable was post-discharge social activity status. Contrarily, the independent variables were those statistically identified as potential predictors in the multiple logistic regression analysis. To regulate the tree size and establish a meaningful minimum size, the tree’s depth was determined to be equivalent to the number of independent variables. The minimum number of cases required for a node was set based on the least frequent category of post-discharge social activity status divided by the maximum permissible node growth. The minimum value on the Gini improvement scale was set at 0.001 to signify a marginal distinction between nodes. A significance level of 5% was used for multiple logistic regression analysis and decision tree analysis. All analyses were conducted using SPSS software for Windows (version 28.0.0.0, IBM Corporation, Armonk, NY, USA).

## 3 Results

Of the 524 patients who underwent OT during hospitalization, 165 who were transferred at discharge or within 1 year of discharge and 112 who were admitted for examination to confirm their psychiatric diagnosis were excluded; 247 were included in the study. Of the patients included in the study, 174 were female, 153 did not have a bachelor’s degree, and 148 had been married; the median age at discharge was 50 years. The most common psychiatric diagnosis was mood disorders (96 patients), followed by others including anxiety disorders (68 patients), schizophrenia spectrum (51 patients), neurodevelopmental disorders (19 patients), and neurocognitive disorders (13 patients). The median GAF score for the entire patient population was 25.

The number of patients who participated in social activities after discharge from the hospital was 188: 63 engaged in employment or academic pursuits, 89 in job seeking or utilization of social services, 5 in self-employment or farming, 4 in voluntary roles, and 27 in hobbies. Of the 116 patients who were socially active at admission, 105 (90.5%) remained socially active after discharge. Contrarily, among patients who were socially inactive at admission, 83 (63.4%) engaged in social activities after discharge: 6 engaged in employment or academic pursuits, 52 in job seeking or utilization of social services, 2 in self-employment or farming, 2 in voluntary roles, and 21 in hobbies.

The total OT participation rates were 31.8%, 38.9%, 43.4%, and 36.8% in the OSP, SAP, AAP, and EP, respectively. Descriptive statistics for the demographic, clinical, and OT-based variables are presented in **Table 1**.

**Table 1:** Characteristics of socially active and socially inactive groups during the first year after discharge.

Factors		Total n = 247	Social activities group n = 188	Non-social activities group n = 59	p-values	
Demographic characteristics						
**Sex** **Age at discharge (years)** **Academic degree** **Marital history** **Social activity status** **at admission**	Male: female median (min-max) University graduate: less than Yes: None Yes: None	73: 174 50.0 (16–88) 87: 153 148: 98 116: 131	63: 125 46.5 (16–88) 71: 113 100: 87 105: 83	10: 49 60.0 (19–83) 16: 40 18: 11 11: 48	0.010 0.006 0.128 < 0.001 < 0.001	* † * * *
Clinical characteristics						
**Diagnostic classifications** **GAF** **Number of** **psychotropic types** **Duration of disorders (years)** **Admission period (days)** **Number of** **past hospitalizations**	ND: SS: MD: MN: Others Median (min-max) Median (min-max) Median (min-max) Median (min-max) Median (min-max)	19: 51: 96: 13: 68 25 (5–45) 2 (0–4) 6.0 (0–48) 57 (3–616) 1 (0–18)	15: 40: 71: 10: 52 25 (10–45) 2 (0–4) 5.5 (0–47) 57 (3–616) 1 (0–18)	4: 11: 25: 3: 16 25 (5–40) 2 (0–4) 8.0 (0–48) 61 (14–115) 2 (1–9)	0.975 0.278 0.774 0.373 0.854 0.529	* † † † † †
Participation ratio in occupational therapy (%)						
**Occupational** **support program** **Seasonal activity program** **Artistic activity program** **Exercise program**	Median (min-max) Median (min-max) Median (min-max) Median (min-max)	31.8 (0.0–100) 38.9 (0.0–100) 43.4 (0.0–100) 36.8 (0.0–100)	38.9 (0.0–100) 41.4 (0.0–100) 44.0 (0.0–100) 38.2 (0.0–100)	12.3 (0.0–90.3) 31.8 (0.0–100) 40.4 (0.0–100) 31.8 (0.0–100)	0.012 0.126 0.435 0.397	† † † †

ND, neurodevelopmental disorders; SS, schizophrenia spectrum; MD, mood disorders; MN, major neurocognitive; Others, anxiety disorders and other included disorders; GAF, Global Assessment of Functioning.

*: Chi-square test.

†: Mann–Whitney U test.

### 3.1 Multiple logistic regression analysis

When the diagnosis of psychiatric disorders was forcibly entered, a stepwise reduction in variables based on preliminary analysis highlighted that marital history (p = 0.003, OR = 0.293, 95% CI 0.130–0.661), social activity status at admission (p < 0.001, OR = 4.557, 95% CI 2.155–9.637), and SAP participation rate (p = 0.015, OR = 1.015, 95% CI 1.003–1.027) were significantly correlated with post-discharge social activity status. Specifically, those who had never been married more than those who had been married, those who participated in social activities more than those who had difficulty at the time of admission, and those who participated more in SAPs were more socially active after discharge. Omnibus Tests of Model Coefficients confirmed statistical significance (p < 0.001). The Hosmer–Lemeshow goodness-of-fit test yielded a value of 0.432, which accurately predicted 77.7% of the outcomes.

### 3.2 Decision tree analysis using the CART algorithm

**Figure 1:**
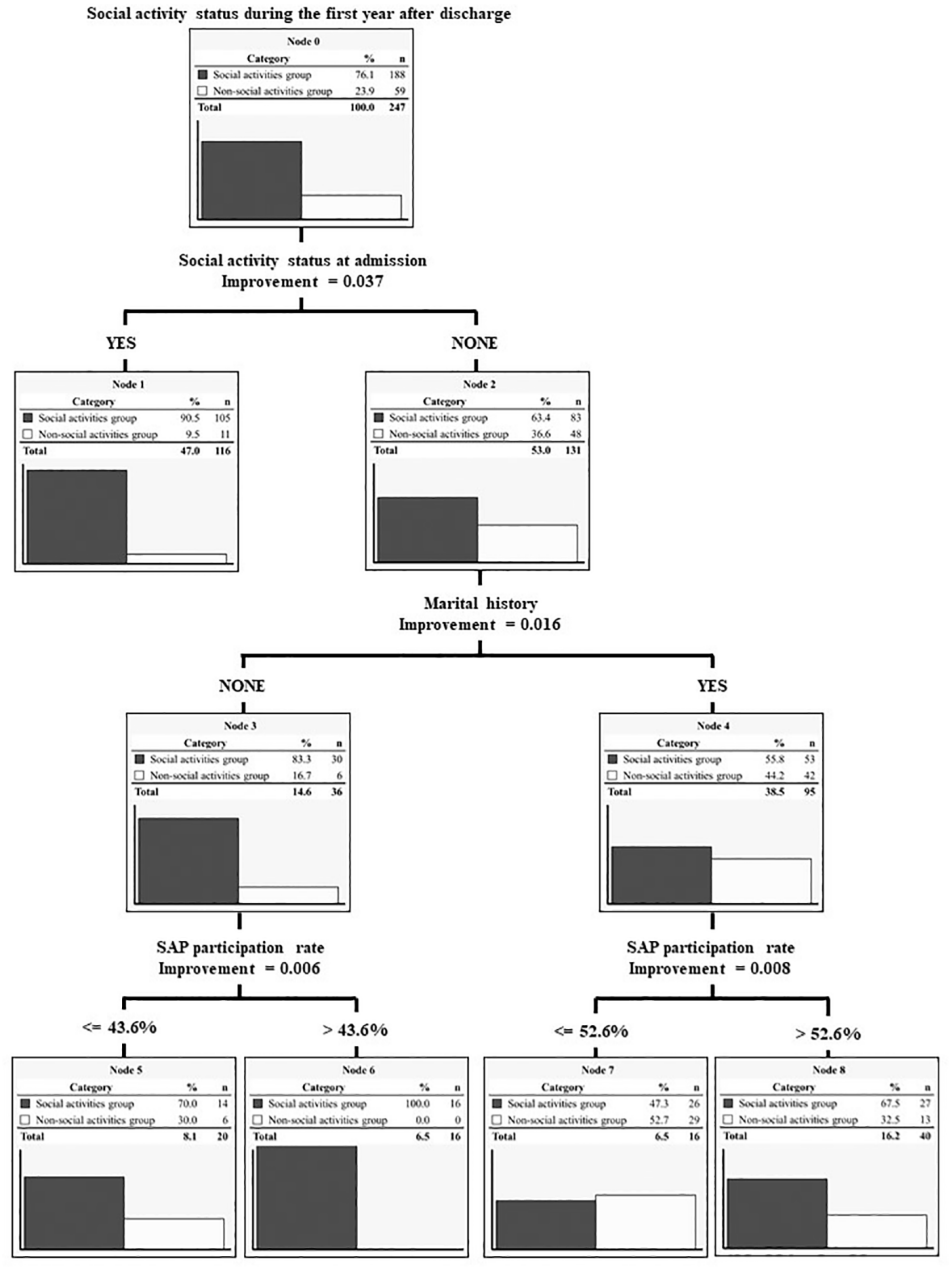
Hierarchy of predictors by the CART algorithm. A hierarchy of three factors extracted by multiple logistic regression analysis is presented. SAP, seasonal activity program (based on OT).

Based on the results of the multiple logistic regression analysis, the decision tree analysis considered marital history, social activity status at admission, and the SAP participation rate as independent variables. We limited the depth of the decision tree to three and established a minimum number of cases per node of 10. The resulting classification and regression tree are illustrated in **Figure 1**. The most influential factor affecting post-discharge social activity was social activity status at admission. Furthermore, among those who were not engaged in social activities at admission, both marital history and SAP participation rates influenced post-discharge social activity status. The SAP participation rate varied according to marital history. All patients who were not involved in social activities at admission and who were never married but participated in 43.6% or more of the SAPs engaged in social activities after discharge. Contrarily, within the node featuring an SAP participation rate of 43.6% or less, six patients still participated in post-discharge social activities.

## 4 Discussion

To the best of our knowledge, this study represents the largest sample size investigation of the determinants influencing post-discharge engagement in social activities for individuals with SMI who received OT during hospitalization in an acute-care university hospital. Furthermore, no other study has used real-world OT practices, which are difficult to populate with a single disorder, to identify the content of OT programs that enable meaningful social participation after discharge and the patient population for whom OT participation needs to be increased. Our findings indicate that OT, particularly programs emphasizing group adaptation, enhances patients’ preparedness for social activities. OT aimed at group adaptation for inpatients can encourage patients to become socially active, especially those admitted in a socially inactive state. Such OT participation rates may vary depending on marital history. The results of this study contribute to the investigation of patient-specific add-on rehabilitation based on the effectiveness of real-world OT practices.

Patients who actively participated in an OT program that facilitated group adaptation were more likely to engage in the defined social activities within the first year of discharge. A crucial element for community reintegration among people with SMI is the development of a sense of community underpinned by feelings of belonging and connectedness (29, 30). Such experiences during hospitalization may be pivotal for post-discharge involvement in social activities. Among inpatients with depression who participated in an OT group using activities, some reported that the activities allowed them to experience “belonging,” “sharing while doing,” and “opening up, which relieved pain” (31). Nevertheless, people with SMI often exhibit insecure attachment styles and face challenges in building trust with others and within themselves (32), which can lead to compromised social functioning (33). To help patients with SMI experience a sense of community during their hospitalization, we decided that conducting very simple occupational activities in a group setting would be effective. The activities introduced in the OT program to facilitate group adaptation were a walk in the hospital garden and seasonal handicrafts. All the patients looked at the same scenery, felt the same wind, and created a work of art together. Collaborative creativity in small groups has been found to reduce barriers to group participation (34), facilitate connections through shared experiences (35), and cultivate a sense of belonging by affirming acceptance within a group of peers with similar experiences (36). Moreover, the structured duration of an OT program that facilitates group adaptation allows participants to build a commitment to the creative process and establish personal expectations for contributing to the project’s completion. These aspects likely enhance patient readiness for social activities by nurturing self-esteem, setting positive expectations about their capabilities, and strengthening their attachment to both their individual endeavors and the community. Such outcomes could contribute to an enduring sense of community following the patients’ return to everyday life after discharge.

Our analysis, employing the CART algorithm, revealed that the impact of participation in an OT program that facilitates group adaptation on post-discharge social activity engagement might vary according to social activity status at admission and marital history. Patients who were able to engage in social activities at admission were not affected by other factors, with 90.5% of patients engaging in social activities after discharge. This result supports Killaspy et al. (14), who showed that pre-admission activity status was associated with successful discharge of patients admitted to a mental health rehabilitation service unit. Talevi et al. (37) also identified factors associated with high social functioning in patients with SMI during hospitalization as not experiencing interpersonal violence, participating in services, and having a high social network index. Social activity status at admission in this study may be similar to the high social network index. Therefore, patients who were participating in social activities at admission in this study may have had higher social functioning and successfully engaged in social activities after discharge without influence from other factors. Among patients who did not participate in social activities at admission, we observed that those who had been previously married were more likely to engage in social activities if they had an SAP participation rate above 52.6%. In contrast, those who had never been married exhibited successful social activity engagement regardless of their participation rate. The link between marriage and mental illness has long been noted (38–40). People with mental illness who are married are more likely to be in poor marriages or to consider divorce (41, 42). In this study, patients who had been married included those who had been divorced. The experience of divorce in patients with SMI has been associated with a fragile attachment style (43) and decreased subjective quality of life (44). Additionally, in the Asia-Pacific region, which includes Japan, traditional and cultural family roles significantly influence access to and provision of care and rehabilitation (45). For this reason, we speculate that to become socially active after discharge, patients with a marital history needed to develop a sense of community by participating in an OT program that facilitated group adaptation more than those who had never been married. Family dynamics can either facilitate or impede recovery in people with SMI (46). Future rehabilitation efforts should balance family involvement with the preferences and needs of people with SMI to improve outcomes. Furthermore, to promote empowerment in patients with SMI and fragile attachment styles, the preliminary establishment of a good therapeutic relationship has been suggested (32). Therefore, to increase OT group participation rates among patients who are unable to participate in social activities at admission and had been married, therapists must first establish a good relationship with their patients and help patients with SMI perceive the therapist as safe.

There are some limitations in interpreting the results of this study. First, the OT programs included groups of heterogeneous and diverse individuals. However, multiple logistic regression analysis indicated that the diagnosis of psychiatric disorders did not affect social activity after discharge. This suggests the existence of important support items that are common across mental illnesses in reconnecting patients with SMI with society. These items may show different aspects for different focal purposes. A good example is the effects of cognitive behavioral therapy conducted with transdiagnostic groups, focused on emotional disorders (47–49). The establishment of purposeful, transdiagnostic group rehabilitation methods in acute psychiatric units for a variety of mental illnesses is significant in terms of healthcare cost reduction. Second, psychoeducation or psychotherapy provided during hospitalization was not considered in the present analysis. It is possible that psychoeducation and psychotherapy provided to individuals may have helped underlie or encourage group OT participation for patients with SMI. Further investigation into the effective sequence and combination of various therapies to support the individual’s wishes is warranted. Third, the social activities identified as outcomes in this study may differ from the individuals’ aimed ultimate recovery. As the journey of recovery from mental illness is an “individual and unique process” (5), it can be difficult to identify the end of the journey during an acute psychiatric unit admission. The results of this study provide suggestions for OT practices that can facilitate the patient’s journey to recovery in an acute psychiatric unit. Finally, the sample size of this study may be insufficient to accommodate decision tree analysis, which is typically a data-mining method for big data. In addition, because this study was conducted at a single site, caution should be exercised in generalizing the results. Further increasing the research data by unifying real-world OT practices at multiple centers and identifying key factors that reconnect SMI patients with society is necessary to examine the methods of transdiagnostic group rehabilitation.

## 5 Conclusion

In this study, we examined the factors contributing to social activity participation 1 year after hospital discharge among patients who received OT during hospitalization at a university hospital providing acute care. The results indicate that encouraging greater participation in OT programs that facilitate group adaptation can improve patient readiness for social activities post-discharge. The findings of this study offer valuable insights into the development of psychiatric rehabilitation content in acute care university hospitals and care expected to be instrumental in assessing the effectiveness of adjunctive therapies.

## Data Availability

The original contributions presented in the study are included in the article/supplementary material. Further inquiries can be directed to the corresponding author.
